# Twelve Tips for Engaging Medical Students in Rural-Focused Research

**DOI:** 10.12688/mep.20642.3

**Published:** 2025-05-16

**Authors:** Grace Perez, Jose Uriel Perez, Aaron Johnston

**Affiliations:** 1 Distributed Learning and Rural Initiatives, University of Calgary, Calgary, Alberta, T2N 4N1, Canada; 2 University of Calgary, Calgary, Alberta, T2N 4N1, Canada

**Keywords:** Rural medicine, rural healthcare interest, undergraduate medical education, student research engagement, research efficacy

## Abstract

**Background:**

The future of rural healthcare depends on training the future rural health workforce, and on rural health research that can guide clinical and policy decisions in rural spaces. Promotion of rural healthcare careers usually focuses on clinical aspects of care, and research may be seen as a lower priority. Supporting students to be involved in rural focused research offers the opportunity to broaden the pool of potentially rural interested students, and to develop research and scholarship skills and capacity in the future rural workforce.

**Aim and method:**

We identify twelve tips that medical schools can adopt to foster medical student participation in rural-focused research and thus promote student interest in rural healthcare and rural medical practice. These recommendations are based on a review of literature and our personal experience of conducting rural-focused research activities with medical students.

**Conclusion:**

Through these twelve tips, we provide a practical framework for enhancing undergraduate medical student exposure to rural-focused research to foster research capacity. This has potential to inspire student interest in future rural medical practice and could contribute to alleviate workforce and research gaps in rural areas.

## Introduction

In the training of medical students, many skills and competencies are deemed necessary for independent practice in healthcare as physicians. While clinical skill development that allows for the highest standard of care of patients is of primary importance during medical training, other components of the training are as vital and contribute to a well-developed physician. One other vital component that needs to be emphasized is the engagement of medical trainees across disciplines in academic and research scholarly activity, which is equally important in medical training and the development of skills ( Bhuiya & Makaryus, 2023). At our institution, the medical curriculum includes opportunities to perform scholarly activities for pre-clinical students. Students often use scholarship as a form of early career exploration. We identified the lack of rural focused scholarly opportunities as a gap in fostering student interest in both rural scholarship, rural training opportunities, and rural careers. Our experience of supporting rural focused scholarly projects has demonstrated interest across diverse domains, from medical education and family medicine, to surgery, planetary health, and artificial intelligence.

Research is essential for the progress of human healthcare, and it is crucial throughout all stages of medical training. At the undergraduate level, research helps introduce pre-clinical students to evidence-based medicine (EBM). Findings from various forms of research have been the cornerstone of evidence-based medicine, informing clinical practice and patient care, health policies and population-based strategies and medical education. Ensuring good health for all people tomorrow depends on future medical research output ( Roberts, 2021). Fostering students’ curiosity and exposure to creative scholarly activities and research training during medical school play a fundamental part in developing physician-scientists who will undertake innovative translational medical research ( Burgoyne *et al.*, 2010). More medical schools are integrating research education and promoting opportunities for research experiences, as means to increase research literacy ( Ward *et al.*, 2024) and ensure that future physician-scientists are armed with skills necessary to pursue research and bridge the fields of science and research ( Muhandiramge *et al.*, 2021).

There are many benefits to undergraduate research involvement for both students and their institutions ( Snow *et al.*, 2010). Engagement in research allows students to explore the effects of applying new thought processes through study and investigation ( Adebisi, 2022). Research experience provides students the chance to develop confidence, think critically, analyze and solve problems, manage time efficiently, and learn collaborative and organization skills that are crucial in any profession ( Kennette & Cappon, 2024; Matthews & Rosa, 2018; Snow *et al.*, 2010). Other benefits of undergraduate research participation have been demonstrated, including increased persistence and retention ( Nagda *et al.*, 1998), improved proficiency in data collection and analysis, and effective speaking and communications skills ( Bauer & Bennett, 2003). These are positively linked to professional advancement and professional and personal development ( Lopatto, 2006; Seymour *et al.*, 2004). Specifically, undergraduate research has proven pivotal at initiating career pathways for minority and underrepresented populations ( Nagda *et al.*, 1998). Other professional benefits of student research participation are the prospect of strong letters of recommendations from research supervisors ( Matthews & Rosa, 2018) and the opportunity of publishing in peer-reviewed journals and presenting at scientific conferences ( Giuliano *et al.*, 2019). Contributions to journals and conferences may afford medical students added advantage in their postgraduate career applications and expand both their personal and institutional visibility in the scientific community ( Adebisi, 2022).

Issues of access and workforce dominate the rural healthcare discourse, and rural focused research may be seen as a lower priority. However, rural focused research is important in delivering care to rural patients whose healthcare needs and context differ from urban patients. Creating necessary rural research capacity requires both the support of interested individuals, and the creation of a positive rural research culture ( Alston *et al.*, 2022; Wong Shee *et al.*, 2022).

While many students articulate interest in research, student involvement varies greatly among medical schools ( Lawson McLean *et al.*, 2013; Muhandiramge *et al.*, 2021) and little is known about the research and scholarly involvement of medical students training in rural communities ( Fox *et al.*, 2023). The authors are from a medical school in western Canada, looking to promote rural healthcare interest and engage students in rural-focused health research. In this paper we present a series of tips that will assist with the implementation of similar initiatives. This includes tips that are specific to rural scholarship as well as more general recommendations about the level of student support required to successfully implement a similar project. This paper is based on the experiences of our department in working with medical students on rural-focused research projects and is informed by a review of relevant literature. One of the authors (JUP) collaborated with our department on a student-led research project and provides a unique student perspective to this paper. Authors GP and AJ provide support to undergraduate medical research in the capacity of research staff and research mentor, respectively.

### Tip 1. Promote awareness and provide opportunities for rural research

The first step to encourage research engagement among students is awareness promotion ( Snow *et al.*, 2010) to inform students of available research opportunities. Medical schools should take resourceful steps to reach out to students and promote opportunities that will help them develop their research skills and passions ( Carberry *et al.*, 2021). At our institution, medical students complete scholarship activities but many are unaware of opportunities for rural experiences, much more so for research activities focused on rural healthcare interest. Our department meets with incoming students during undergraduate medical orientation and other student-information events. For instance, during *Rural Health Week*, a week-long rural-oriented campaign for rural healthcare interest, we make sure to inform students what our department offers regarding research opportunities, including a research funding grant discussed in Tip 11. We are also considering having a student advocate to speak at this event, who has previously worked with our research team, to share their first-hand experiences, making the opportunities more relatable and inspiring to their peers. Additionally, we have a dedicated section on our department website that outlines the application process for the research grant funding opportunities and how to get involved in research opportunities. We are expanding this to include a list of student-led ongoing and completed research projects and research interests of faculty members and community preceptors. This online resource could also include success stories and testimonials from students ( Snow *et al.*, 2010).

While medical students may have individual motivation for research involvement, not every student may be interested in undertaking an entire rural research project. When students reach out to us, we make time to meet them and discuss the best way we can help and nurture their interest. We include in the discussion the different types of research projects and the different extents of participation required. We also encourage students to initiate and create research ideas and generate opportunities for themselves ( Mabvuure, 2012). From our experience, students usually already have some good ideas about a research topic. For such instances, we help in focusing their research questions and objectives, fine-tune their study proposals and guide them on the appropriate research approaches and methodologies discussed in Tip 5. For students looking for join an ongoing research project, we may integrate them into current research teams within our department or encourage them to collaborate with other researchers within our institution. We find that it is helpful to ask other faculty members and preceptors engaged in independent research if they would like to have students as part of their research team ( Snow *et al.*, 2010).

### Tip 2. Inspire passion and purpose and encourage students to look at a ‘rural perspective’

From our experience, our department’s enthusiasm for rural research attracts medical students to engage in collaborative research with us. Describing the value of research for their learning and career goals is another way to inspire students to pursue research ( Al-Busaidi, 2020; Mabvuure, 2012). For instance, undergraduate research engagement offers career-oriented benefits in “enhancement of professional or academic credentials” and “clarification of a career path” ( Lopatto, 2003). Further, students engaged in research absorb the culture of research (embracing high standards of scientific integrity, disseminating novel discoveries and assisting other researchers) and are more likely to reap the positive outcomes described above ( Russell *et al.*, 2007). In keeping with our mission for rural medical education, when engaging with students, we endeavor to raise the profile of rural research within the institution and encourage them to look at a ‘rural perspective’ as part of their research ( Alston *et al.*, 2022). We use the opportunity to express the relevance of EBM in rural clinical practice settings ( Lawson McLean *et al.*, 2013). We advocate for rural healthcare interest and put the spotlight on ‘rural context’, by including a rural component as one of the research questions. In doing this, we aim to help students keep the rural perspective present in their minds and hopefully inspire them to consider rural medical practice as part of their future career path.

### Tip 3. Cultivate relationships with rural interest clubs and other student societies

From our experience, student societies, particularly rural interest clubs, are valuable resources for engaging a cohort of students who share similar interests. These student organizations can offer opportunities for students to develop interest in research and to attend educational events aimed at developing skills relevant to research. Exposure to such events has been shown to increase medical students’ interest in research ( Lawson McLean *et al.*, 2013). Cultivating strong relationships with student groups can significantly enhance the visibility and interest in rural-focused research opportunities. These groups often have interdisciplinary backgrounds, with many medical students having a variety of undergraduate degrees. This diversity in student scholarly backgrounds can bring fresh perspectives to research projects, and the research becomes more robust and inclusive ( Skorinko, 2019). Each student's unique experiences and strengths contribute to a richer, more comprehensive approach to research, enabling teams to address problems from multiple angles. Further, as the students progress in their academic studies, their group roles are passed on to a new cohort of students, facilitating an opportunity to initiate new projects or continue with current and existing research.

### Tip 4. Facilitate meaningful connections between students and rural physicians and focus on rural physicians’ strengths as knowledge experts

At our institution most of the time in preclinical training is spent in an urban environment at a centralized medical school. Although this period, early in training, is ideal for involvement with research for rurally interested students, the students often lack connections to rural physicians to assist in developing their projects. Likewise, rural physicians in our setting are often very busy with clinical roles and clinical teaching and lack the time to independently supervise the entirety of a scholarly project and may not conceptualize research as a central part of their role. Bridging this gap is an important component of a successful student project ( Zorzi *et al*., 2005).

Linking students to rural physicians provides critical context for student projects. Rural physicians are experts in their environments and in the challenges of rural medicine. Making these connections early in a project allows students to refine their questions and ensures that project outcomes are meaningful and connected to actual rural issues. Care must be taken that the rural physicians are also supported in this role, taking on the tasks that they are interested in and have time for. Practically we have found that many rural physicians thrive when acting as knowledge experts, consulting on a project. Providing central support for administrative tasks, like ethics submissions, and data analysis, and emphasizing the rural physician’s expertise in defining the research problem and providing contextual information has been a successful strategy for us in facilitating meaningful connections between rurally interested students and rural physicians.

Some concrete examples of this approach include: an ongoing project focused on rural simulation, and ongoing project focused on the role of rural narratives ( Malhi *et al*., 2023; Perez *et al*., 2024), and a completed project focused on peer-led learning ( Perez *et al*., 2025). Each of these projects has facilitated connections between students interested in rural research, and rural physicians. Although some of these rural physicians have limited prior experience in research, they have been able to meaningfully participate and support students, by focusing on their strengths.

### Tip 5. Conceptualize scholarship broadly, as both small and large projects have value in rural spaces

Both students and rural physicians may have misconceptions about ‘what counts’ as research and scholarship, but as Boyer's model of scholarship ( Boyer, 1990) has pointed out, scholarship is not limited to research alone, and encompasses the broader spectrum of scholarly activities that contribute to knowledge creation, application, and the improvement of teaching and learning within medical education. In our experience rural physicians often take a scholarly approach to solving local problems, though they may not disseminate their solutions to other rural physicians and sites and often don’t conceptualize this work as scholarship. Likewise, students may have preconceived notions about research and scholarship focusing on the types of projects they have been exposed to during premedical studies such as wet labs, and large research teams within universities. At the project development stage students require assistance in defining research question, methods, and project scope. These choices must be informed by the realities of the time that students and rural physicians are able to dedicate to a project, resources available to support the project, and the size and experience of the project team.

Emphasizing the use of the scholarly method as an approach to the challenges of rural medicine sets up learners for long term success whether the projects they take on are big or small. Some students will take on large projects, for example an ongoing project on the impact of rural emergency department closures, and a completed scoping review on rural surgical access ( Ebrahim *et al*., 2025). These types of projects align with traditional ideas about scholarship and are disseminated in traditional ways, such as conference presentations and publications. Other students have the time and expertise to take on a small project in the course of their training. An example of a small project is the identification of a specific challenge within a rural placement, and conducting a focused literature review, and the development of a poster for knowledge translation. This process mirrors how practicing clinicians can approach the challenges of rural practice in a scholarly way. These types of focused local projects may require support for dissemination as they may be considered too local for publication. We have found that creating a forum for sharing these types of projects at one of our rural conferences has been very successful and generated significant interest among rural physicians. Past examples of this type of poster project include: increasing understanding of local Indigenous issues, supporting communication with neurodivergent youth in the primary care setting, practical tips on handwashing in the clinic, and promoting movement as medicine.

### Tip 6. Provide support for fundamental elements of research

Both students and rural physicians need support for fundamental research activities in order to successfully complete projects ( Zorzi *et al*., 2005). Both groups face substantial time pressure and may have limited experience in various elements of research. Even when students or rural physicians do have experience, the nuances of institutional protocols around ethics submission, access to statistical software, etc. may be a frustrating challenge. In order to foster rural research a balance of support and teaching is required across a variety of research skills and domains.

Fundamental research methods and processes are part of most undergraduate curriculum ( Adebisi, 2022; Giuliano *et al.*, 2019). Yet many medical students may still struggle with coherent methods and lack an approach for formal study design ( McKelvie & Standing, 2018). Our department reinforces their research education by providing students the opportunities to apply the lessons to practice. Many student ideas usually lean towards investigations of relationships and interventional research. Being mindful of the timeframe required to complete the research, we guide students on writing proposals that justify using methods for quantitative research and qualitative research that have manageable timelines. Understandably, medical students are looking to finish the research before their program completion. Hence, ideal research projects would have a relatively short data gathering, such as inquiries that involve readily available public health data and other data sources, or studies utilizing cross-sectional surveys, interviews, focus groups and knowledge synthesis from literature reviews. Students may still be involved in longer-term projects such as multi-stage studies, where their participation could be tapped for specific stages or phases of an ongoing research project.

One hurdle that medical students engaging in research face is the consideration of the appropriate data procedures. A lack of solid foundation for statistics and research design can hamper student research engagement ( Mendoza & Matone, 2019). Many students grapple with appropriate statistical methods and sufficient sample sizes for adequate statistical power ( McKelvie & Standing, 2018). Students often lack the capacity and have less access to resources to conduct the relevant data analysis ( Matthews & Rosa, 2018). Our department offers students with expert support on software for qualitative analysis (e.g. NVivo), quantitative analysis (e.g. SPSS, Stata) and assistance in setting up their literature reviews on reference manager software such as Covidence and EndNote. Our research team takes charge of the more complex statistical analysis and data modelling that may be required, and guides students in the thematic coding during qualitative data analysis. We also help prepare data summary tables for poster presentations and manuscript submissions.

### Tip 7. Foster research literacy

Involving medical students in research stimulates their creativity and helps foster research self-efficacy in the long run ( Adebisi, 2022). In our department, we encounter students with different levels of research capacity. Some are experienced and already have PhD or MSc degrees. Some are novices and must be guided accordingly. Recognizing the different levels of experience of our students, we meet the students where they are ( Skorinko, 2019). To increase their research literacy, it is important to include students from research planning to execution ( Lawson McLean *et al.*, 2013). We rely on our rural expertise and innate enthusiasm to guide the rural-focused research projects. We consider medical students as research collaborators to help them take ownership ( Skorinko, 2019) and we work as a team during the research process from inception to completion. We work with students in focusing their research questions, defining the study objectives, preparing the study proposal, establishing study timelines, collecting and analyzing data and writing up the research findings. For research involving human participants we help students navigate the intricate ethics submission and review process. We also developed a library of practical tools and resources such templates for writing the study proposals and abstracts, designing research posters and creating research slide presentations and grant funding application.

### Tip 8. Connect students to the research community and resources as needed

We found it useful to expose and connect students to the wider research community within the institution ( Lawson McLean *et al.*, 2013). Some projects may need a subject matter expert to act as principal investigator or research mentor, while others may need collaboration with other interdisciplinary teams and resources such as the university librarian and data repository team. For example, when students wanted to synthesize the evidence about rural access to surgery, we directed them to work with a university librarian to conduct a systematic and comprehensive search of literature from relevant scientific journal databases. Similarly, when students sought to study the impact of rural emergency department closures, we connected them to the data owners of provincial health system databases to retrieve relevant information to answer their research question. Our faculty members and preceptors also help facilitate research engagement by sharing their own ongoing research with students. Notably, faculty members and preceptors can provide much-needed research mentorship. Our department sends out a monthly newsletter to faculty members and preceptors and we regularly ask them for interest to be mentors. Mentorship in research is an important support for fostering students to become the next generation of research scientists ( Lawson McLean *et al.*, 2013), providing structure and clear expectations ( Giuliano *et al.*, 2019), and navigating challenges and problem-solving during the research ( Lawson McLean *et al.*, 2013). For our team, mentoring includes determining novel areas for research, finding scientific conferences and journals for students to present their work and assisting in manuscript preparation, specifically in offering valuable insight and context on the intricate nuances of implication to practice of research findings.

### Tip 9. Set realistic expectations and monitor the research progress

From our experience, it is important to set realistic expectations and timelines. Medical students often juggle demanding schedules, and research projects can sometimes be sidelined or delayed as a result. To keep projects from falling behind, it is essential to not only mentor students but also actively monitor their progress and provide ongoing support to keep their projects on track. In addition, establishing a diverse research team can be particularly effective ( Skorinko, 2019). By including students from different programs or academic years, who have varying schedules, the workload can be more evenly distributed. This way, when some students are occupied with exams or clinical responsibilities, others can step in to keep the project moving forward. Further, we found it important to offer guidance on realistic timelines, as students may not fully understand the time required for tasks such as data collection, interpretation, manuscript writing, and submission for publication. By sitting down with our students to set clear, achievable goals, we help ensure that students remain focused and meet their objectives. This approach not only fosters collaboration but also helps students develop time management and teamwork skills, which are invaluable in their future careers ( Lopatto, 2010; Matthews & Rosa, 2018; Skorinko, 2019).

### Tip 10. Encourage and support students to publish and present their work

It is advantageous for medical students to present their work at seminars, scientific meetings and conferences ( Adebisi, 2022; Bhuiya & Makaryus, 2023; Mabvuure, 2012). We encourage students to attend conferences where they can showcase their work, such as the *Rural and Remote Medicine Conference*. Our department also annually hosts an in-house professional development conference, *Cabin Fever,* where we regularly invite students to present their work in a friendly environment. Outside our department, there are also several other scientific meetings, seminars and symposia within the institution where we encourage our students to exhibit their research work as applicable. Presenting at conferences not only allows students to share their work, it helps increase self-confidence and improve their presentation skills ( Bauer & Bennett, 2003). Additionally, it can attract more institutional research collaborations ( Adebisi, 2022), and result in increased engagement in research among students ( Little, 2020; Mass-Hernandez *et al.*, 2022). In addition to presenting at conferences, we support students to publish their work ( Lawson McLean *et al.*, 2013). Contributions to scientific conferences and peer-reviewed journal can bolster medical students’ CVs with measurable outcomes and their subsequent applications for medical residency programs ( Bhuiya & Makaryus, 2023). There are scientific journals (e.g., *International Journal of Medical Students*, *Canadian Journal of Undergraduate Research*, etc.) that are specifically meant for publication of undergraduate research output. It is helpful to let students know that it may require submissions to multiple journals before the article is published. Because research does not always guarantee publishable results, we also help students to consider other channels to communicate research findings such as a policy briefs, reports and white papers, websites and traditional media. It is also important to emphasize that publishing is a bonus to the knowledge gained in research self-efficacy ( Mabvuure, 2012).

### Tip 11. Invest in research funding

Our institution actively invests in advancing knowledge creation and dissemination. Our department provides grant funding for rural-focused research projects. Moreover, we fund and pay for a dedicated fulltime research associate position which coordinates our department’s research activities, and provides vital methodological, data analysis and writing support for student research projects. As further discussed in Tip 12, we also provide funding for students to present their work at scientific conferences, which allows them to learn from the experiences of other researchers ( Adebisi, 2022), and other research capacity building initiatives at the institutional level. Our funding can also be used to cover publication costs, especially fees to publish in open-access journals which provide greater visibility to the research work. As discussed in Tip 1, we include in our website a section on the research grant funding to clearly communicate the funding opportunity to students, including grant amounts and the allowable expenses covered.

### Tip 12. Celebrate student success and raise institutional reputation as a student-friendly program

 We believe that success leads to more success, so we actively celebrate students’ accomplishments in rural-focused research. Inviting our student researchers to present and share their work at our internal conference, *Cabin Fever*, serves as a chance to laud their contributions to advance rural healthcare knowledge among the rural community of educators, physician-scientists and peers. We also give incentives such as travel grants to scientific conferences and support for publication fees ( Adebisi, 2022). We believe that apt recognition strengthens students’ feelings of motivation and achievement. Moreover, in helping medical students build their research capacity and commending their success, our department is also steadily gaining a reputation as a student-friendly program, which is a boon for our mission in rural medical education. This can establish close and frequent contact between more students, faculty members and research team, enabling students to be more motivated and actively involved in learning ( Komarraju *et al.*, 2010), which in turn can build more enthusiasm for more research collaborations ( Skorinko, 2019). To strengthen student-faculty relationships and showcase the benefits of collaboration, it is helpful to publicize successful past projects with different student groups, ideally by providing a list of previous student projects, on the institution’s website. Highlighting these projects not only demonstrates the variety of research activities available but also serves as a powerful testimonial to the outcomes achieved through collaboration.

## Conclusion

The underlying goal of engaging students in rural-focused research is to concomitantly promote rural healthcare interest while building research aptitude. Student engagement in rural-focused research bolsters and reinforces rural medical education and mutually benefits both students and the institution. Students gain research self-efficacy that enables them to explore solutions to real-world problems. It also provides other avenues for rural preceptors to teach, serve and conduct research. This generates institutional enthusiasm about rural research across domains and fosters the creation of scholars within the rural community and ultimately propels the underlying mission of rural medical education. Rural research engagement has potential to inspire student interest in future rural medical practice and could contribute to alleviate the physician shortage in rural areas.

## Ethics and consent

Ethical approval and consent were not required.

## Data Availability

No data are associated with this article.

## References

[ref-5] Adebisi YA : Undergraduate students' involvement in research: values, benefits, barriers and recommendations. *Ann Med Surg (Lond).* 2022;81: 104384. 10.1016/j.amsu.2022.104384 36042923 PMC9420469

[ref-19] Al-Busaidi IS : Medical student journals: promoting academic research and publishing. *New Zealand Medical Student Journal.* 2020; (30):48–49. 10.57129/CHFL4276

[ref-15] Alston L Field M Brew F : Addressing the lack of research in rural communities through building rural health service research: establishment of a research unit in Colac, a medium rural town. *Aust J Rural Health.* 2022;30(4):536–539. 10.1111/ajr.12860 35254718 PMC9544785

[ref-10] Bauer KW Bennett JS : Alumni perceptions used to assess undergraduate research experience. *J High Educ.* 2003;74(2):210–230. 10.1080/00221546.2003.11777197

[ref-30] Bhuiya T Makaryus AN : The importance of engaging in scientific research during medical training. *Int J Angiol.* 2023;32(3):153–157. 10.1055/s-0042-1759542 37576537 PMC10421692

[ref-31] Boyer EL : Scholarship reconsidered: priorities of the professoriate. ERIC,1990. Reference Source

[ref-2] Burgoyne LN O'Flynn S Boylan GB : Undergraduate medical research: the student perspective. *Med Educ Online.* 2010;15(1): 5212. 10.3402/meo.v15i0.5212 20844608 PMC2939395

[ref-22] Carberry C McCombe G Tobin H : Curriculum initiatives to enhance research skills acquisition by medical students: a scoping review. *BMC Med Educ.* 2021;21(1): 312. 10.1186/s12909-021-02754-0 34078364 PMC8173745

[ref-32] Ebrahim A Sinha S Adedipe I : Rurality predisposes departure from gold-standard care, leading to delayed or accelerated access to surgery: insights from a scoping review. *Can J Surg.* 2025;68(1):E17–E31. 10.1503/cjs.000124 39753325 PMC11684926

[ref-17] Fox JL Cribb J Cumming K : Medical student interest and participation in research at one rural clinical school: insights from the last six years. *Aust J Rural Health.* 2023;31(3):569–574. 10.1111/ajr.12970 36762881

[ref-13] Giuliano T Skorinko JLM Fallon M : Engaging undergraduates in publishable research: best practices. *Front Psychol.* 2019;10:1878. 10.3389/fpsyg.2019.01878 31507477 PMC6719508

[ref-8] Kennette LN Cappon A : Student interest in research roles: barriers and benefits. *J Appl Res Community Coll.* 2024;31(1):53–70. Reference Source

[ref-29] Komarraju M Musulkin S Bhattacharya G : Role of student–faculty interactions in developing college students’ academic self-concept, motivation, and achievement. *J Coll Stud Dev.* 2010;51(3):332–342. 10.1353/csd.0.0137

[ref-16] Lawson McLean A Saunders C Velu PP : Twelve tips for teachers to encourage student engagement in academic medicine. *Med Teach.* 2013;35(7):549–554. 10.3109/0142159X.2013.775412 23496123

[ref-27] Little C : Undergraduate research as a student engagement springboard: exploring the longer-term reported benefits of participation in a research conference. *Educ Res.* 2020;62(2):229–245. 10.1080/00131881.2020.1747360

[ref-20] Lopatto D : The essential features of undergraduate research. *CUR quarterly.* 2003;24:139–142. Reference Source

[ref-12] Lopatto D : Undergraduate research as a catalyst for liberal learning. *Peer Rev.* 2006;8(1). Reference Source

[ref-25] Lopatto D : Undergraduate research as a high-impact student experience. *Peer Rev.* 2010;12(2). Reference Source

[ref-18] Mabvuure NT : Twelve tips for introducing students to research and publishing: a medical student's perspective. *Med Teach.* 2012;34(9):705–709. 10.3109/0142159X.2012.684915 22905656

[ref-33] Malhi R Perez G Shafiq J : Practical tips for using a Human Library approach in medical education [version 1; peer review: 1 approved, 2 approved with reservations]. *MedEdPublish.* 2023;13:208. 10.12688/mep.19746.1 38371395 PMC10873544

[ref-28] Mass-Hernández LM Acevedo-Aguilar LM Lozada-Martínez ID : Undergraduate research in medicine: a summary of the evidence on problems, solutions and outcomes. *Ann Med Surg (Lond).* 2022;74: 103280. 10.1016/j.amsu.2022.103280 35127067 PMC8807964

[ref-7] Matthews SJ Rosa MN : Trials, tribulations, and triumphs: research and publishing from the undergraduate perspective. *Front Psychol.* 2018;9:2411. 10.3389/fpsyg.2018.02411 30559700 PMC6286968

[ref-24] McKelvie S Standing LG : Teaching psychology research methodology across the curriculum to promote undergraduate publication: an eight-course structure and two helpful practices. *Front Psychol.* 2018;9:2295. 10.3389/fpsyg.2018.02295 30538651 PMC6277486

[ref-26] Mendoza SA Martone LE : From the classroom to the lab: how faculty can extend curriculum oriented research experiences to publish with undergraduates. *Front Psychol.* 2019;10:622. 10.3389/fpsyg.2019.00622 30971979 PMC6445845

[ref-4] Muhandiramge J Vu T Wallace MJ : The experiences, attitudes and understanding of research amongst medical students at an Australian medical school. *BMC Med Educ.* 2021;21(1): 267. 10.1186/s12909-021-02713-9 33971858 PMC8108334

[ref-9] Nagda BA Gregerman SR Jonides J : Undergraduate student-faculty research partnerships affect studen retention. *Rev High Educ.* 1998;22(1):55–72. 10.1353/rhe.1998.0016

[ref-34] Perez G Groves A Fehr L : Peer-led Learning: a novel approach to promote rural healthcare interest among medical students. * Front Med (Lausanne).* 2025;12: 1566472. 10.3389/fmed.2025.1566472 40357284 PMC12066776

[ref-35] Perez G Malhi R Bressler K : Challenging perceptions about rural practice using narratives: a living library approach in medical education. *Front Med (Lausanne).* 2024;11: 1452932. 10.3389/fmed.2024.1452932 39720659 PMC11667788

[ref-1] Roberts LW : Going extraordinary distances with physician–scientists. *Acad Med.* 2021;96(4):477–478. 10.1097/ACM.0000000000003923 33782227

[ref-21] Russell SH Hancock MP McCullough J : The pipeline. Benefits of undergraduate research experiences. *Science.* 2007;316(5824):548–549. 10.1126/science.1140384 17463273

[ref-11] Seymour E Hunter AB Laursen SL : Establishing the benefits of research experiences for undergraduates in the sciences: first findings from a three-year study. *Sci Educ.* 2004;88(4):493–534. 10.1002/sce.10131

[ref-23] Skorinko JLM : Looking back at undergraduate research experiences to promote the engagement of undergraduates in publishable research at an R2 institution. *Front Psychol.* 2019;10: 1316. 10.3389/fpsyg.2019.01316 31214100 PMC6558172

[ref-6] Snow AA de Cosmo J Shokair SM : Low-cost strategies for promoting undergraduate research at research universities. *Peer Rev.* 2010;12(2). Reference Source

[ref-3] Ward B Diug B Wallace M : Immersed in scholarly projects: upskilling our future medical workforce. *Focus on Health Professional Education: A Multi-Professional Journal.* 2024;25(1):78–87. 10.11157/fohpe.v25i1.719

[ref-14] Wong Shee A Quilliam C Corboy D : What shapes research and research capacity building in rural health services? Context matters. *Aust J Rural Health.* 2022;30(3):410–421. 10.1111/ajr.12852 35189009 PMC9304287

[ref-36] Zorzi A Rourke J Kennard M : Combined research and clinical learning make rural summer studentship program a successful model. *Educ Health (Abingdon).* 2005;18(3):329–37. 16236581 10.1080/13576280500289330

