# Supplementary material for: Twelve Tips for Engaging Medical Students in Rural-Focused Research
Source: MedEdPublish (2016). 2025 May 16;14:243. Originally published 2024 Oct 22. [Version 3] doi: 10.12688/mep.20642.3 (PMC11809146; doi:10.12688/mep.20642.3)
Supplement: Peer review history

# Reviewer report: Reviewer response for version 3

**DOI:** 10.21956/mep.22569.r41832

**Published:** 2025-06-03

**Authors:** Fury Maulina

**Affiliations:** 1 Department of Public Health, Faculty of Medicine, Malikussaleh University (Ringgold ID: 175452), Lhokseumawe, Aceh, Indonesia

This revised version has undergone significant improvements. The twelve tips provided will be helpful, especially in promoting/advancing research on rural healthcare—an area that remains frequently under-studied. This article may also further inspire medical schools, especially those with a focus on rural medical education, to develop and adapt their curricula tailored to their contextual and distinctive features. 

If evidence from practice is presented, are all the underlying source data available to ensure full reproducibility?

Not applicable

Is the topic of the practical tips discussed accurately in the context of the current literature

Yes

Are all factual statements correct and adequately supported by citations?

Partly

Are the conclusions drawn balanced and justified on the basis of the presented arguments?

Yes

Are arguments sufficiently supported by evidence from the published literature and/or the authors’ practice?

Yes

Reviewer Expertise:

Physician leadership development in underserved areas, such as rural and remote settings, and how cultural factors influence (physician) leadership practice in rural and remote health systems.

I confirm that I have read this submission and believe that I have an appropriate level of expertise to confirm that it is of an acceptable scientific standard.

# Reviewer report: Reviewer response for version 3

**DOI:** 10.21956/mep.22569.r41831

**Published:** 2025-05-28

**Authors:** James Rourke

**Affiliations:** 1 Memorial University of Newfoundland, St. John's, Canada

The revised report is now more rural-focused, with additional rural-specific tips. It is a practical addition to the literature and will be helpful to rural students, rural physicians, and medical school rural research teams.

If evidence from practice is presented, are all the underlying source data available to ensure full reproducibility?

Partly

Is the topic of the practical tips discussed accurately in the context of the current literature

Yes

Are all factual statements correct and adequately supported by citations?

Yes

Are the conclusions drawn balanced and justified on the basis of the presented arguments?

Partly

Are arguments sufficiently supported by evidence from the published literature and/or the authors’ practice?

Yes

Reviewer Expertise:

NA

I confirm that I have read this submission and believe that I have an appropriate level of expertise to confirm that it is of an acceptable scientific standard.

# Reviewer report: Reviewer response for version 2

**DOI:** 10.21956/mep.22306.r40645

**Published:** 2025-01-16

**Authors:** Fury Maulina

**Affiliations:** 1 Department of Public Health, Faculty of Medicine, Malikussaleh University (Ringgold ID: 175452), Lhokseumawe, Aceh, Indonesia

This is an interesting article that mentions twelve 'practical' tips for engaging medical students in conducting rural-focused research. This article would also be valuable for educational leaders and other relevant stakeholders in medical schools/education to encourage research that focuses on rural contexts and issues. However, I have a few suggestions accordingly. 

 1. In the conclusion section (abstract), I would suggest using the word ‘tips’ instead of ‘framework’.

 2. In the abstract, the authors mentioned the method they applied to this paper. I would suggest providing methods as well in the main paper (how they provided literature and/or explored authors' experiences). 

 3. The authors highlighted the importance of conducting rural-focused research, however, very limited literature was provided describing the rural context of why rural issues should be investigated especially in the Canadian context. It would be interesting for international readers to know the challenges that occur in the rural context of Canada and what makes the challenges distinct from other similar countries like Canada (to attract the readers).

 4. I found that tips 1 and 2 overlap. Can authors provide the main point of each tip of those two if they have a different point of view? 

 5. The conclusion is a bit long. I suggest making it even more concise, and the last sentence mentioned: "... to alleviate the physician shortage in rural areas", seems the authors didn't mention about this issue.

If evidence from practice is presented, are all the underlying source data available to ensure full reproducibility?

Not applicable

Is the topic of the practical tips discussed accurately in the context of the current literature

Yes

Are all factual statements correct and adequately supported by citations?

Partly

Are the conclusions drawn balanced and justified on the basis of the presented arguments?

Yes

Are arguments sufficiently supported by evidence from the published literature and/or the authors’ practice?

Yes

Reviewer Expertise:

Physician leadership development in rural or remote contexts

I confirm that I have read this submission and believe that I have an appropriate level of expertise to confirm that it is of an acceptable scientific standard, however I have significant reservations, as outlined above.

# Reviewer report: Reviewer response for version 2

**DOI:** 10.21956/mep.22306.r40731

**Published:** 2025-01-13

**Authors:** James Rourke

**Affiliations:** 1 Memorial University of Newfoundland, St. John's, Canada

This article speaks to the importance of student research in medical education. It offers tips for engaging medical students in rural-focused research based on an excellent research program.

 As this paper is based on one institution’s experience, it would be helpful for the international audience to have more context about the relevant rural-urban geography, the stage of learners, and the Canadian system, which is generally postgraduate entry into medical school and 3-4 years of medical school.  

 The article uses the phrase “From our Experience” several times. It would be helpful for the article to list the experiences on which they base this. How many students (and what percentage of total medical students) participated in Rural Research projects? How many went on to publish? This could be provided as part of the added context in the introduction.  

 I agree with Dr. Hogenbirk’s and Dr. McGrail's review comments that many of the tips as currently written, are not necessarily unique to rural research. Much of the introduction relates to the general benefits of medical student research. Most of the readers of this article will be interested in engaging medical students in rural-focused research. Cutting out much of the general student research content would make room for more detailed rural-specific tips. This could include more on navigating the ethics required for some research projects, including Indigenous research relationships; who takes long-term responsibility for the project’s completion and knowledge translation; working with and supporting rural healthcare providers along with the students to do rural research. Some key issues we faced 20 years ago are still challenging (see RRH 2005 reference).

If evidence from practice is presented, are all the underlying source data available to ensure full reproducibility?

Partly

Is the topic of the practical tips discussed accurately in the context of the current literature

Yes

Are all factual statements correct and adequately supported by citations?

Yes

Are the conclusions drawn balanced and justified on the basis of the presented arguments?

Partly

Are arguments sufficiently supported by evidence from the published literature and/or the authors’ practice?

Yes

Reviewer Expertise:

Rural Medical Education, Rural Health Care

I confirm that I have read this submission and believe that I have an appropriate level of expertise to confirm that it is of an acceptable scientific standard, however I have significant reservations, as outlined above.

## References

[1] : Combined research and clinical learning make Rural Summer Studentship Program a successful model. *Rural Remote Health* .2005;5(4) :401 16283822

# Reviewer report: Reviewer response for version 2

**DOI:** 10.21956/mep.22306.r40732

**Published:** 2025-01-03

**Authors:** Jean Kerver

**Affiliations:** 1 Michigan State University, East Lansing, Michigan, USA

This paper, written for the “Practical Tips” section of the MedEdPublish publishing platform, describes 12 tips for engaging medical students in rural-focused research that were developed by the authors after their own experiences mentoring medical students at a medical school in western Canada. Fostering research in medical education with a focus on rural populations is an important endeavor for all the reasons listed in the introduction section of the paper and the tips are sound. I have only minor comments as noted below.

  

1. Because this is accessible to an international audience, it would help to clarify what is meant by undergraduate vs. pre-clinical undergraduate vs. medical education. The introduction refers to all these stages, yet the focus of the paper is medical education. I am confused and I suspect other readers may be confused, too. Please consider clarifying, by editing the introduction section, if you are suggesting that students should begin rural-focused research during their undergraduate education, i.e., before they even begin medical school, or during medical school. And, if pre-clinical undergraduate education is part of medical school, perhaps a description of the stages of medical education in Canada is warranted.
2. In addition to the reasons noted by the authors for promoting rural-focused research, other reasons are that by having had their own research experiences, physicians in rural areas are better equipped to understand the literature that guides their practice, and it helps them make clinical observations in a systematic way that may lead to new discoveries. The authors may wish to incorporate these ideas somewhere in their tips.

If evidence from practice is presented, are all the underlying source data available to ensure full reproducibility?

Not applicable

Is the topic of the practical tips discussed accurately in the context of the current literature

Yes

Are all factual statements correct and adequately supported by citations?

Yes

Are the conclusions drawn balanced and justified on the basis of the presented arguments?

Yes

Are arguments sufficiently supported by evidence from the published literature and/or the authors’ practice?

Yes

Reviewer Expertise:

Perinatal Epidemiology; Nutrition; Rural Health Disparities

I confirm that I have read this submission and believe that I have an appropriate level of expertise to confirm that it is of an acceptable scientific standard.

# Reviewer report: Reviewer response for version 2

**DOI:** 10.21956/mep.22306.r40529

**Published:** 2024-12-21

**Authors:** John Hogenbirk

**Affiliations:** 1 NOSM University Postgraduate Education Office (Ringgold ID: 432413), Sudbury, Ontario, Canada

Thank you for the opportunity to review “Twelve Tips for Engaging Medical Students in Rural-Focused Research”. The manuscript proposes twelve tips that medical schools can adopt to encourage medical student participation in rural-focused research, with the complementary goals of promoting student interest in rural healthcare and rural medical practice. These are laudable goals with reasonable evidence linking them and this paper would do well to help promote these outcomes.

 I agree with Dr. McGrail's comment in the first review that many of the tips, as currently written, are not necessarily unique to rural research. Although the authors note that "rural focused research is important in delivering care to rural patients whose healthcare needs and context differ from urban patients", and speak of capacity building in rural research, their first point assumes that "opportunities for rural research" already exist and that urban-based researchers have sufficient expertise and contextual knowledge. A "Tip 0" might be to "engage and support rural research and rural-based researchers" (as per Alston et al. 2022).

 Supporting rural-based researchers is important because very often it is urban-based researchers (including me) who are conducting rural research and it behoves us to involve rural-based researchers and rural-based research partners in substantive roles during project conception, implementation, analysis, interpretation, and dissemination (Wong Shee et al. 2012). This is of particular importance when research involves Indigenous people, marginalized groups, other vulnerable populations, or groups under-represented in research. Acknowledging that it is the responsibility of the principal researcher or mentor and that medical students follow their lead, it is nonetheless important that rural people be involved in meaningful ways right from the start. The authors mention some of this in the conclusion "It also provides other avenues for rural preceptors to teach, serve and conduct research" but offer minimal support in the rest of the paper. For instance, the examples in Tip 8 could refer to projects in which rural people helped inform, shape, interpret, and disseminate rural research projects.

 In regards to methods, mention should be made of the unique challenges of conducting rural research. Challenges such as mistrust of urban-based researchers, doubts as to whether urban-based researchers really understand the rural context (Wong Shee et al. 2012), varying participation rates, small sample sizes/small area variation, missing data, irrelevancy of some administrative data to actual service delivery and use (Wong Shee et al. 2012), difficult and expensive travel for on-site data collection, and so forth. Rural populations often have, for example, higher disease burdens, lower social economic status, and more dangerous occupations, which affect health needs and health outcomes—all of these can influence how research is conducted, interpreted and used.

 Also acknowledging that it is the responsibility of the principal researcher or mentor, more could be said about the importance of ethical approval. The current mention: "For research involving human participants we help students navigate the intricate ethics submission and review process", could be reworded and enlarged to emphasize the role of ethical approval in improving research rather than as an administrative barrier to be crossed. Research ethics is of great importance when one group (urban-based researchers) conducts research on another group (rural people).

 The paper could benefit from a definition of "rural", perhaps in comparison with "urban", with the caveat that not all rural areas and rural populations are the same. For instance, rural populations in cottage areas with seasonal and permanent residents as well as tourists, may or may not resemble adjacent rural areas. In addition, some rural areas host retirement villages, while people in other rural areas commute to work in nearby urban areas—the people and infrastructure in these rural areas can, for example, differ greatly from the people and infrastructure in agricultural or resource-extraction areas.

 The paper could also benefit from a definition of research. For example, do "research" projects include evaluations, Continuous Quality Improvement projects, policy reviews, etc.? Does research include qualitative and quantitative methods? If agreeable, it should suffice for the authors to write that "we take a broad view of research that includes [examples of eligible types] employing qualitative and/or quantitative methods suitable to the study question". In addition, the phrase at the end of the second paragraph of the introduction is perplexing ("bridge the fields of science and research"). What is the difference between science and research that the authors wish to bridge?

 There seems to be some overlap in several of the tips. "Opportunities" mentioned in Tip 2 is part of Tip 1. Additionally, Tip 2 addresses the MATCH of the student to the project and perhaps should be renamed "matching student to opportunity". Some material from Tip 7 (research literacy) could be moved to the renamed Tip 2. Importance of rural clubs in Tip 4 could be included with Tip 3 (passion, rural perspective) or in Tip 1 (Awareness). Tips 5-8 all speak to research methods (none unique to rural research): Tips 5 and 7 could be easily combined and mention of timelines in Tip 5 can be removed as it is covered in Tip 9; Tip 8 "Connect students to research community" is already part of Tip 2 (renamed). Funding of students is mentioned in Tips 10, 11, and 12.

 There are a few typographical errors that need correction, both are noted by Dr. Greenhill in an earlier review. These include changing "student involvement vary" to " students' involvement varies"; and changing "as well as guides students in the thematic coding" to "and guide students in the thematic coding".

 Some of my comments refer to the student project in the broader context of the medical school rural research program. Nonetheless, acknowledgement of the broader context is important to ensure that rural research conducted by medical students reflects more of the needs of rural populations and addresses local concerns (Wong Shee et al. 2012). This approach does not negate the expertise that can be housed in urban centers, instead it explicitly recognizes local and contextual expertise that can help make research more impactful and meaningful.  

 Respectfully submitted,

 John C. Hogenbirk

 Associate Director, Centre for Rural and Northern Health Research - Laurentian University

 Family Medicine Scholarly Activity Tutor, Northern Ontario School of Medicine University

If evidence from practice is presented, are all the underlying source data available to ensure full reproducibility?

Yes

Is the topic of the practical tips discussed accurately in the context of the current literature

Partly

Are all factual statements correct and adequately supported by citations?

Yes

Are the conclusions drawn balanced and justified on the basis of the presented arguments?

Partly

Are arguments sufficiently supported by evidence from the published literature and/or the authors’ practice?

Yes

Reviewer Expertise:

Rural and remote health services; rural and remote health human resources; rural and remote medical residents scholarly activity (research) projects.

I confirm that I have read this submission and believe that I have an appropriate level of expertise to confirm that it is of an acceptable scientific standard, however I have significant reservations, as outlined above.

# Response

**Published:** 2025-05-21

**Authors:** Grace Perez

**Affiliations:** Distributed Learning and Rural Initiatives, University of Calgary, Calgary, Alberta, Canada

Dear Dr. Hogenbirk, Thank you for your time to review our manuscript, version 2, and for providing us your valuable feedback to make improvements.

 We have submitted an updated version (version 3) of the manuscript, incorporating changes to reflect most of your suggestions, together with suggestions from Dr. Rourke and Dr. Maulina. 

 With much appreciation,

 Grace Perez and Aaron Johnston

# Reviewer report: Reviewer response for version 2

**DOI:** 10.21956/mep.22306.r40440

**Published:** 2024-12-20

**Authors:** Matthew R McGrail

**Affiliations:** 1 The University of Queensland, Rockhampton, Queensland, Australia

I have no further comments to add. Approved

If evidence from practice is presented, are all the underlying source data available to ensure full reproducibility?

Yes

Is the topic of the practical tips discussed accurately in the context of the current literature

Yes

Are all factual statements correct and adequately supported by citations?

Yes

Are the conclusions drawn balanced and justified on the basis of the presented arguments?

Partly

Are arguments sufficiently supported by evidence from the published literature and/or the authors’ practice?

Partly

Reviewer Expertise:

All parts of the paper

I confirm that I have read this submission and believe that I have an appropriate level of expertise to confirm that it is of an acceptable scientific standard.

# Response

**Published:** 2024-12-20

**Authors:** Grace Perez

**Affiliations:** Distributed Learning and Rural Initiatives, University of Calgary, Calgary, Alberta, Canada

Dear Dr. McGrail, Thank you for your time to review both versions of our paper. We truly appreciate your support and your helpful comments to improve our paper.  Warmly, Grace Perez

# Reviewer report: Reviewer response for version 1

**DOI:** 10.21956/mep.22097.r40360

**Published:** 2024-11-30

**Authors:** Jennene Greenhill

**Affiliations:** 1 Nursing, Southern Cross University (Ringgold ID: 110940), Gold Coast, Queensland, Australia; 2 Southern Cross University, Adelaide, Australia

Thanks for the opportunity to review this paper. I agree with the authors rural research builds capacity and attracts people to rural areas but I think it is important to add that research builds intellectual capital in rural areas. Also rural research is often multidisciplinary and therefore it is important to say that it strengthens interprofessional approaches to healthcare that are so vital in rural communities. This comes in Tip 8 but should also be motioned in the introduction.

 In the sentence While many students articulate interest in research, student involvement vary, please change vary to varies.

 Change each of the paragraphs beginning with "From our experience to introduce the tip i.e. To foster students to have a rural perspective... To cultivate student's interest ... Realistic expectations are important because...

 In Tip 6 change "as well as guides" to "and guides"

 Tip 11 should include how research funding contributes to strengthening the university's international profile, which adds to a long-term sustainable commitment to strengthening our rural communities. There is a mutual benefit for rural communities and universities through investing in research and development. 

 In the conclusion change to "mutually benefits students, rural health professionals, the institutions both health services and universities and the wider most of all benefits patients and wider rural communities. 

 The above could reference Paul Worley's symbiotic model which includes research as a fundamental arm of the model.

If evidence from practice is presented, are all the underlying source data available to ensure full reproducibility?

Yes

Is the topic of the practical tips discussed accurately in the context of the current literature

Yes

Are all factual statements correct and adequately supported by citations?

Yes

Are the conclusions drawn balanced and justified on the basis of the presented arguments?

Yes

Are arguments sufficiently supported by evidence from the published literature and/or the authors’ practice?

Yes

Reviewer Expertise:

rural health, health professional education. aged care

I confirm that I have read this submission and believe that I have an appropriate level of expertise to confirm that it is of an acceptable scientific standard.

# Response

**Published:** 2024-12-20

**Authors:** Grace Perez

**Affiliations:** Distributed Learning and Rural Initiatives, University of Calgary, Calgary, Alberta, Canada

Dear Greenhill, thank you for taking the time to review our paper.  Warmly, Grace Perez

# Reviewer report: Reviewer response for version 1

**DOI:** 10.21956/mep.22097.r39566

**Published:** 2024-11-11

**Authors:** Matthew R McGrail

**Affiliations:** 1 The University of Queensland, Rockhampton, Queensland, Australia

This 12 tips paper is a refreshing addition to the literature. Note that I directly supervised Fox et al 2023 with her key evidence in undertaking research during rural medical training, which attempted to quantify how often such activity led onto published outcomes. Notably, that was in a context of MD students completing at least 1 (sometimes 2) years of their clinical training ‘in-place’ within rural communities.

 However, in this new 12 tips paper, I was unclear of the context they are centring this on. Negligible mention is made of any of the students actually spending time in rural communities or rural healthcare facilities. Can the authors please clarify? If I have this incorrect, please add information to the paper and how the tips are targeted to address this. If I have this correct, I’m surprised this is not directly addressed in any of the tips or background. Either way, more context information is needed.

 A few parts of the Abstract are misleading / need editing. In the background, I found the first 1.5 lines to be simplistic / overly obvious (i.e. having more healthcare depends on training more healthcare). The aim has a key phrase missing (“rural-focused”). Rewrite as: We identify twelve tips that medical schools can adopt to foster medical student participation in RURAL-FOCUSED research and THUS promote student interest in rural healthcare and rural medical practice. In the conclusion, I don’t understand why the first sentence ends with “to foster research capacity”…but that’s a secondary outcome, not their aim.

 In the Introduction, it is notable that zero parts of the first 2 paragraphs relate to rural health / rural context. I’d encourage the authors to integrate this much earlier, given its key focus to the rationale of their study.

 Focusing on the 12 tips, my biggest concern is that many of the tips are somewhat generic to place / context (i.e. just as applicable to metro-based medical students and fostering research). In tip 1, the header notes “opportunities for ‘rural’ research, but the only fleeting mention of rural relates to one example event (rural health week). I was expecting this to be more centred on growing rural research opportunities (noting that earlier there is no mention of these students being ‘in place’ of rural communities. Many of the tips have zero direct rural connection (e.g. #5, 6, 9, 10). I urge the authors to edit these so that it is clearer how they specifically engage / foster more RURAL research. As they are presented currently, these read like tips to support increased med student research in any context (e.g. large metropolitan).

If evidence from practice is presented, are all the underlying source data available to ensure full reproducibility?

Yes

Is the topic of the practical tips discussed accurately in the context of the current literature

Yes

Are all factual statements correct and adequately supported by citations?

Yes

Are the conclusions drawn balanced and justified on the basis of the presented arguments?

Partly

Are arguments sufficiently supported by evidence from the published literature and/or the authors’ practice?

Partly

Reviewer Expertise:

All parts of the paper

I confirm that I have read this submission and believe that I have an appropriate level of expertise to confirm that it is of an acceptable scientific standard, however I have significant reservations, as outlined above.

# Response

**Published:** 2024-12-03

**Authors:** Grace Perez

**Affiliations:** Distributed Learning and Rural Initiatives, University of Calgary, Calgary, Alberta, Canada

**Reviewer comments:** This 12 tips paper is a refreshing addition to the literature. Note that I directly supervised Fox et al 2023 with her key evidence in undertaking research during rural medical training, which attempted to quantify how often such activity led onto published outcomes. Notably, that was in a context of MD students completing at least 1 (sometimes 2) years of their clinical training ‘in-place’ within rural communities.

***Response: **We are grateful for your time to review our paper. We value your insights and helpful comments.*

**Reviewer comments: **However, in this new 12 tips paper, I was unclear of the context they are centring this on. Negligible mention is made of any of the students actually spending time in rural communities or rural healthcare facilities. Can the authors please clarify? If I have this incorrect, please add information to the paper and how the tips are targeted to address this. If I have this correct, I’m surprised this is not directly addressed in any of the tips or background. Either way, more context information is needed.

***Response: **Thank you for your comments. We have added a paragraph in the introduction to provide more context to our paper.*

**Reviewer comments: **A few parts of the Abstract are misleading / need editing. In the background, I found the first 1.5 lines to be simplistic / overly obvious (i.e. having more healthcare depends on training more healthcare). The aim has a key phrase missing (“rural-focused”). Rewrite as: We identify twelve tips that medical schools can adopt to foster medical student participation in RURAL-FOCUSED research and THUS promote student interest in rural healthcare and rural medical practice. In the conclusion, I don’t understand why the first sentence ends with “to foster research capacity”…but that’s a secondary outcome, not their aim.

***Response: **We agree with your suggested edits and have re-written and corrected the abstract. Thank you.*

**Reviewer comments: **In the Introduction, it is notable that zero parts of the first 2 paragraphs relate to rural health / rural context. I’d encourage the authors to integrate this much earlier, given its key focus to the rationale of their study.

***Response: **We have added a paragraph in the introduction to provide more context to our paper.*

 Focusing on the 12 tips, my biggest concern is that many of the tips are somewhat generic to place / context (i.e. just as applicable to metro-based medical students and fostering research). In tip 1, the header notes “opportunities for ‘rural’ research, but the only fleeting mention of rural relates to one example event (rural health week). I was expecting this to be more centred on growing rural research opportunities (noting that earlier there is no mention of these students being ‘in place’ of rural communities. Many of the tips have zero direct rural connection (e.g. #5, 6, 9, 10). I urge the authors to edit these so that it is clearer how they specifically engage / foster more RURAL research. As they are presented currently, these read like tips to support increased med student research in any context (e.g. large metropolitan).

***Response: **Thank you for your helpful and constructive comments.* *For Tip 1, we have added a statement to provide more context that* *at our institutio*n, medical students complete scholarship activities but many are unaware of opportunities for rural experiences, much more so for research activities focused on rural healthcare interest. And thus our department tries to encourage student interest in research activities and also rural healthcare. For Tips #5, 6#, #9, #10, we agree that these tips are applicable to any medical student research in any interest area or context, but we feel it is also important to those who want to consider embarking of rural research. These tips cover topics that are key considerations for anyone considering setting up a rural-focused research program. Although these tips may seem obvious, there is a significant paucity of rural research in our location (Canada) and universities considering similar initiatives need to have a clear idea of the level of support and resources required to successfully implement this type of project.  

 With much appreciation,

 Grace Perez and Aaron Johnston
